# RANKL expression in chondrocytes and its promotion by lymphotoxin-α in the course of cartilage destruction during rheumatoid arthritis

**DOI:** 10.1371/journal.pone.0254268

**Published:** 2021-07-07

**Authors:** Ayumu Takeshita, Keiichiro Nishida, Aki Yoshida, Yoshihisa Nasu, Ryuichi Nakahara, Daisuke Kaneda, Hideki Ohashi, Toshifumi Ozaki

**Affiliations:** Department of Orthopaedic Surgery, Okayama University Graduate School of Medicine, Dentistry and Pharmaceutical Sciences, Okayama, Japan; Polytechnic University of Bucharest, ROMANIA

## Abstract

We investigated the expression and localization of the receptor activator nuclear factor κB ligand (RANKL) in cartilage from patients with rheumatoid arthritis (RA) of relevance to cartilage degeneration. We also examined the role of exogenous lymphotoxin (LT)-α on RANKL expression in human chondrocytes and its effect on *in vitro* osteoclast differentiation. Cartilage and synovial fluid samples were obtained from 45 patients undergoing total joint replacement surgery or joint puncture, including 24 patients with osteoarthritis (OA) and 21 patients with RA. RANKL expression in articular cartilage was examined by immunohistochemistry. LT-α concentrations in synovial fluid were measured using an enzyme-linked immunosorbent assay (ELISA). Normal human chondrocytes were stimulated with LT-α, and the relative mRNA levels of RANKL, osteoprotegerin (OPG), matrix metalloproteinase-9, and vascular endothelial growth factor were examined by real-time polymerase chain reaction. Soluble RANKL protein in culture media was measured using ELISA, and membrane-bound RANKL protein in cells was examined by western blotting. Co-cultures of human chondrocytes with peripheral blood mononuclear cells (PBMCs) were stimulated with macrophage-colony stimulating factor and LT-α, and osteoclast differentiation was evaluated by staining for tartrate-resistant acid phosphatase. LT-α concentrations were higher in RA synovial fluid than in OA samples. The population of RANKL-positive chondrocytes of RA cartilage was higher than that of OA cartilage, and correlated with cartilage degeneration. Stimulation of cultured human chondrocytes by LT-α increased RANKL expression, the RANKL/OPG ratio, and angiogenic factors. Membrane-bound RANKL in chondrocytes was up-regulated after stimulation of LT-α, whereas soluble RANKL in culture medium did not increase. Co-cultures of human chondrocytes and PBMCs demonstrated that LT-α stimulated human chondrocytes to produce RANKL and induced osteoclastic differentiation of PBMCs. RANKL produced by chondrocytes may contribute to cartilage destruction during RA and LT-α could promote the expression of RANKL in human chondrocytes.

## Introduction

Rheumatoid arthritis (RA) is a chronic inflammatory disease with unknown etiology and is characterized by bone and cartilage destruction [1]. Recent advancements in pharmacologic therapies, including inhibitors for tumor necrosis factor (TNF)-α, interleukin (IL)-6 and Janus kinases, T cell co-stimulation blockers, and B cell depletion agents significantly improve the disease state and functional impairment of RA patients [2]. However, approximately 30–40% of patients either do not achieve an adequate response to therapy or become resistant to such treatments [3].

The receptor activator of nuclear factor κB ligand (RANKL) is a key molecule for the differentiation and activation of osteoclasts [4, 5], and is involved in the bone resorption of inflammatory joint diseases, such as RA. A recent study demonstrated that the serum concentrations of RANKL were increased before RA onset [6], and were associated with anti-citrullinated protein antibodies in early untreated RA [7]. A study of early and active RA patients also showed that the higher the RANKL/OPG ratio one year after the onset of the disease correlated with the more progressive joint destruction of the hands and feet, as evaluated by the Sharp/van der Heijde score [8].

RANKL is synthesized as a trans-membrane molecule in numerous cell types in bone and joints, including osteoblasts, osteocytes, synovial fibroblasts, T cells, and B cells [9]. RANKL expression in chondrocytes was first reported in studies of embryonic mouse long bones, and its expression was maintained throughout the development [10]. Komuro *et al*. examined RANKL expression in normal and mild osteoarthritic (OA) cartilage, and found that RANKL was expressed in chondrocytes of the superficial and midzone, as well as in cells clustered in OA cartilage [11]. More recently, Moreno-Rubio *et al*. examined RANKL expression and localization in advanced OA cartilage, and reported expression in most cells in all zones. Interestingly, that study described RANKL localization in the pericellular area in the deep zone, tidemark-embedded chondrocytes, and some chondrocytes in calcified cartilage [12]. However, there is scarce information about the expression and localization of RANKL in cartilage of patients with RA.

The results of immunohistochemistry analyses of the joint tissues of antigen-induced arthritis (AIA) model rabbits exhibited higher expression of RANKL than in normal and OA cartilage. Diffuse RANKL distribution was observed throughout the articular cartilage [13]. Thus, we aimed to investigate RANKL expression and localization in cartilage samples from patients with RA.

Several molecules involved in RA pathogenesis have been reported to stimulate RANKL expression in chondrocytes. Some studies have suggested that TNF-α, IL-1β, and prostaglandin (PG)E_2_ induce the expression of RANKL in human chondrocytes [11, 13, 14]. Lymphotoxin-α (LT-α; TNF-β) has strong homology and similar biological activity to TNF-α. Additionally, LT-α is predominantly produced by lymphocytes and involved in the maintenance of the immune system, including the development of secondary lymphoid organs [15]. LT-α is only expressed in a soluble homotrimeric form and is also involved in the regulation of cell survival, proliferation, differentiation, and apoptosis [16]. Increased levels of LT-α have been reported in serum and synovial tissues from patients with RA, compared to healthy individuals or patients with OA [17]. In addition, LT-α was found to induce cell proliferation at a similar level to TNF-α in cultured fibroblast-like synovial cells (FLS) from RA patients. However, it is unknown whether LT-α plays a role in the regulation of RANKL expression in human chondrocytes.

In the latter part of this study, we investigated the *in vitro* effects of LT-α on the expression of RANKL and osteoprotegerin (OPG) in cultured human chondrocytes. It was revealed that LT-α stimulated RANKL expression in chondrocytes and promoted the differentiation of osteoclast-like cells. Thus, it is possible that LT-α-induced RANKL expression in chondrocytes may have a role in joint destruction during RA independently of TNF-α.

## Materials and methods

### Preparation of clinical samples

The current study received the approval of our Institutional Review Board (Okayama University No. 1607–036). Patients were included from Okayama University Hospital in Japan and provided written informed consent prior to participation between July 2016 and March 2018. Experiments were conducted at the department of orthopaedic surgery, Okayama University Graduate School of Medicine, Dentistry and Pharmaceutical Sciences. We calculated sample size with power analysis before the start of the study, if the effect size of OA samples was 0.50, that of RA samples was 0.20, the detection power was 85%, and the significance level was 1%, required sample size was 64. However, clinical samples were actually obtained from 45 patients and it was not possible to obtain more samples during the recruitment period. As the number of samples decreased, it was decided to change the significance level 1% to 5% to improve the statistical power.

Clinical samples were obtained from 45 patients undergoing total joint replacement of the knee or metacarpophalangeal joints, or joint puncture of the knee or shoulder joints during daily practice. Twenty four patients (M/F: 4/20) aged 49–83 years who were diagnosed with OA according to ACR criteria [18], and 21 patients (M/F: 4/17) aged 35–77 years who were diagnosed with RA according to ACR criteria [19] were included in the study (Table 1). None of the RA patients received TNF inhibitors, and four RA patients received tocilizumab, an anti-IL-6 receptor antibody. The synovial fluid samples were obtained from 11 knee joints from 11 patients with OA (11 samples), and one shoulder joint and nine knee joints from 10 patients with RA (10 samples) (Table 1). The articular cartilage samples were obtained from six tibial plateaus and seven femoral condyles from 13 patients with OA (13 samples), two tibial plateaus and 11 metacarpal heads from 11 patients with RA (13 samples) (Table 2). Articular cartilage samples were fixed in 10% formalin solution and then decalcified in 20% ethylenediaminetetraacetic acid (EDTA) before embedding in paraffin blocks. Synovial fluid used for LT-α and TNF-α protein assays were immediately centrifuged at 2000 rpm for 10 minutes to remove debris, and hyaluronidase (Sigma, St. Louis, MO) was added at a concentration of 100 μg/ml. The samples were incubated at 37°C for 20 minutes, then centrifuged at 8000 rpm for 10 minutes to remove the cells. Synovial fluid samples were stored at -80°C until examination.

**Table 1:** Individual patient data and concentrations of LT–α and TNF–α in synovial fluid samples.

Case No.	age	Gender	Disease duration (years)	Medication	DAS28-CRP	Joint	LT-α (pg/ml)	TNF-α (pg/ml)
RA 1	66	F	47	MTX	3.25	Shoulder	16.75	800.18
RA 2	76	M	1	TCZ, MTX, SASP, PSL	3.65	Knee	102.14	1147.42
RA 3	73	F	27	MTX, PSL	4.24	Knee	6.91	15.16
RA 4	71	M	4	TCZ, MTX, PSL	3.28	Knee	6.65	6.57
RA 5	64	F	17	MTX, TAC, PSL	2.44	Knee	20.37	31.3
RA 6	75	F	51	none	1.92	Knee	4.60	13.41
RA 7	72	F	8	none	2.18	Knee	5.79	25.18
RA 8	60	F	6	TAC, SASP	3.37	Knee	6.69	5.83
RA 9	50	M	6	SASP, PSL	N/A	Knee	8.47	27.22
RA 10	64	F	41	MTX	4.41	Knee	4.60	5.83
OA 1	69	M				Knee	6.08	5.50
OA 2	68	F				Knee	4.60	5.50
OA 3	70	F				Knee	4.60	5.50
OA 4	70	F				Knee	9.32	5.50
OA 5	81	F				Knee	4.60	8.21
OA 6	61	F				Knee	4.60	5.50
OA 7	71	F				Knee	13.32	66.63
OA 8	72	F				Knee	4.60	5.50
OA 9	82	M				Knee	4.60	5.50
OA 10	82	F				Knee	4.60	15.75
OA 11	49	F				Knee	4.60	13.41

The detection limit of the LT–α ELISA kit was 4.6 pg/ml, and that of the TNF–α ELISA kit was 5.5 pg/ml. The values below the detection limit were taken as detection limit values. DAS28–CRP, disease activity score 28–joint count C reactive protein; PSL, prednisolone; MTX, methotrexate; TAC, tacrolimus; SASP, salazosulfapyridine; TCZ, tocilizumab; BUC, bucillamine; N/A, not available.

**Table 2:** Individual patient data and Mankin’s score and RANKL expression ratio in articular cartilage samples.

Case No.	sample No.	age	Gender	Disease duration (years)	Medication	Operation	Mankin’s score	RANKL expression ratio (%)
RA 11	1	35	F	17	MTX, PSL	FJA	4	32.5
	2						5	31.5
RA 12	3	77	M	10	MTX, BUC	FJA	9	57.3
	4						9	51.3
RA 13	5	68	F	26	MTX, PSL	FJA	8	42.9
RA 14	6	66	F	6	MTX	FJA	7	41.9
RA 15	7	63	F	33	MTX, PSL	FJA	8	37.7
RA 16	8	62	F	23	MTX, SASP, PSL	FJA	9	47.2
RA 17	9	76	F	5	TCZ	TKA	5	33.2
RA 18	10	65	F	10	MTX, PSL	TKA	8	45.1
RA 19	11	41	F	30	PSL	FJA	9	53.3
RA 20	12	74	F	24	MTX, PSL	FJA	8	49.3
RA 21	13	71	F	31	TCZ, PSL	FJA	8	47.2
OA 12	1	69	M			TKA	9	46.9
OA 13	2	67	F			TKA	8	20.9
OA 14	3	73	F			TKA	7	17.7
OA 15	4	67	F			TKA	8	11.7
OA 16	5	58	F			TKA	7	20.4
OA 17	6	74	F			TKA	8	35.7
OA 18	7	79	F			TKA	5	11.1
OA 19	8	83	M			TKA	7	29.2
OA 20	9	77	F			TKA	6	4.6
OA 21	10	66	F			TKA	5	4.4
OA 22	11	78	F			TKA	8	11.3
OA 23	12	68	F			TKA	8	29.7
OA 24	13	66	F			TKA	8	22.5

The RANKL expression ratio was determined by dividing the number of chondrocytes positive for RANKL by the total number of chondrocytes. The value was the average of three fields of view. DAS28–CRP, disease activity score 28–joint count C reactive protein; PSL, prednisolone; MTX, methotrexate; TAC, tacrolimus; SASP, salazosulfapyridine; TCZ, tocilizumab; BUC, bucillamine; FJA, finger joint arthroplasty; TKA, total knee arthroplasty.

### Histological evaluation of cartilage destruction

Safranin O/fast green stain was performed on articular cartilage, and we evaluated the severity of joint destruction in each joint using the histologic histochemical grading system described by Mankin *et al*. [20]. The Mankin system assesses four parameters, including the structure of articular cartilage (0–6), the cellularity of chondrocytes (0–3), proteoglycan staining (0–4), and the irregularity of tide marks (0–1). The scores are summed to determine normal cartilage (Score 0) to severe degeneration (Score 14).

### Immunohistochemical evaluation of RANKL in articular cartilage and RANKL expression ratio in chondrocytes

Deparafinized cartilage sections were pretreated with 0.1 M citrate buffer (pH 6.0) in an autoclave at 90°C for 5 minutes to retrieve the antigen. Rabbit anti-RANKL antibodies (dilution, 1:500; cat. no. ab9957; Abcam, Cambridge, UK) were used as primary antibodies at 4°C overnight. Histofine^®^ Simple Stain Rat MAX PO (R) (Nichirei Biosciences, Tokyo, Japan) was used as the secondary antibody. The reaction was visualized by diaminobenzidine, and counterstaining was carried out with hematoxylin. Sections incubated with non-immune rabbit serum were used as negative controls. RANKL expression in articular chondrocytes was assessed as the RANKL expression ratio, which was determined by dividing the number of chondrocytes positive for RANKL by the total number of chondrocytes. The value was the average of three fields of view. The magnification when obtaining images was set to x100 to cover the entire layer of articular cartilage. In case it was difficult to judge the presence or absence of RANKL expression, the judgement was made at a higher magnification.

### Immunoelectron microscopy

Cartilage specimens were fixed in 0.1 M phosphate-buffered 4% paraformaldehyde, washed with 0.1 M phosphate-buffered saline (PBS, pH 7.4) and decalcified by EDTA. Samples were then embedded in a hydrophilic resin (LR-White), and subjected to ultrathin sectioning. After treatment with 6 M urea for 5 minutes and blocking non-specific binding using 0.1 M PBS containing 1% bovine serum albumin (BSA) and 10% goat serum, sections were treated for overnight at 4°C with the rabbit polyclonal anti-human RANKL antibodies (primary antibodies; cat. no. ab9957; Abcam) at 2 μg/ml. Control specimens were treated with blocking solution without primary antibodies. After washing with PBS, samples were treated with 0.48 μg/ml goat polyclonal anti-rabbit IgG (H+L) conjugated with 10 nm colloidal gold (BBI solutions, Ltd, UK) as the secondary antibody for 1.5 hours at room temperature. Samples were washed with 0.1 M PBS containing 1% BSA, fixed with 2% glutaraldehyde for five minutes, stained with uranyl acetate and lead citrate, and examined under a transmission electron microscope (Hitachi, H-7650, Central Research Laboratory, Okayama University Medical School).

### Cells and cell culture

Chondrocytes derived from normal human knee joint cartilage obtained from a 6-year-old female (lot no. 6F4018), 34-year-old male (lot no. 8F3339), and 45-year-old male (lot no. 8F3304) were purchased from Lonza (Walkersville, MD, USA). All cell lines were provided virus (human immunodeficiency virus, hepatitis B virus, and hepatitis C virus) testing, mycoplasma testing, and cell viability testing. Cells were cultured in chondrocyte basal medium (CBM^TM^) (Lonza) containing chondrocyte growth medium (CGM^TM^) BulletKit^TM^ (Lonza), 10% fetal bovine serum, recombinant human fibroblast growth factor-β, long *R*^3^ insulin-like growth factor, transferrin, insulin, gentamicin, and amphotericin-B at 37°C in the presence of 5% CO_2_. The medium was changed every two days, and human normal articular chondrocytes were used at passage three or four. The cells were seeded onto type I collagen-coated 6-well plates at a density of 1.5 × 10⁵ cells/well. Cells were cultured to 80% confluence in CBM^TM^ with the CGM^TM^ BulletKit^TM^, and transferred to CBM^TM^ with 1% fetal bovine serum for 24 hours before exposure to stimuli. Then, chondrocytes were stimulated with LT-α (R&D Systems, Minneapolis, MN, USA) (0, 1 or 10 ng/ml) or TNF-α (R&D Systems) (0, 1 or 10 ng/ml). Cells were stimulated for 12 or 24 hours for mRNA determination and for 48, 60 or 72 hours for protein determination. In other experiments, cells were preincubated with biological DMARDs, 10 ng/ml etanercept (Pfizer Japan Inc, Tokyo, Japan) or 10 ng/ml adalimumab (Eisai Co., Ltd., Tokyo, Japan), for 30 minutes before stimulation with LT-α or TNF-α. Etanercept inhibited both LT-α and TNF-α, while adalimumab inhibited only TNF-α.

### Real-time PCR analysis

Cells were washed twice with PBS at 12 and 24 hours after cytokine stimulation, and total RNA was isolated using the RNeasy^®^ Mini Kit (Qiagen, Hilden, Germany). RNA samples were reverse-transcribed using ReverTra Ace (Toyobo, Osaka, Japan). The resulting cDNAs were used for real-time polymerase chain reaction (PCR) amplification. Real-time PCR was performed using an Mx3000P QPCR System (Agilent Technologies, Santa Clara, CA, USA) with TaqMan Gene Expression Assays for human RANKL (Hs00243522_m1), OPG (Hs00900358_m1), matrix metalloproteinase (MMP)-9 (Hs00957562_m1), vascular endothelial growth factor (VEGF) (Hs00900055_m1), and GAPDH (Hs03929097_g1) (Applied Biosystems, Foster City, CA, USA). Amplification of the reference gene, GAPDH, was used to normalize the efficiency of cDNA synthesis and the concentration of RNA. We calculated the final expression levels by dividing the expression levels of RANKL, OPG, MMP-9, and VEGF by the expression level of GAPDH. Each value obtained for the control cells (chondrocytes without stimulation) was set to one.

### Western blot analysis

Expression of membrane-bound RANKL was detected by western blot analysis. Chondrocytes were resuspended in Mammalian Protein Extraction Buffer (GE Healthcare, Piscataway, NJ, USA) containing protease inhibitors (diluted 1:100, Takara Bio USA, CA, USA) 60 hours after cytokine stimulation. Protein concentrations were determined by the Bradford method. Similar amounts of protein extracts were loaded onto sodium dodecyl sulfate–polyacrylamide gels (Any kD^TM^ Mini-PROTEAN^®^ TGX^TM^ Gels (Bio-Rad, Munchen, Germany)) and run for 45 minutes at 150 V before transfer to polyvinylidene difluoride membranes using a Trans-Blot^®^ Turbo^TM^ Blotting System (Bio-Rad). The membranes were incubated with blocking reagent (Toyobo) and incubated overnight at 4°C with RANKL antibodies (dilution, 1:500; cat. no. ab9957; Abcam). After washing with washing buffer, the membranes were incubated with IRDye Goat anti-Rabbit IgG (LI-COR Biosciences, Lincoln, NE, USA) secondary antibodies at room temperature for 1 hour. Immunoreactive proteins were detected using the OdysseyFc Imaging System (LI-COR Biosciences). We analyzed the densities of the resulting protein bands using the OdysseyFc Imaging System. The levels of membrane-bound RANKL were expressed as ratios and normalized to β-actin.

### ELISA for LT-α and TNF-α in synovial fluid, and soluble RANKL and OPG in the culture medium

The concentrations of LT-α and TNF-α in synovial fluid collected from RA and OA patients were measured using enzyme-linked immunosorbent assay (ELISA) kits for LT-α (cat. no. NBP1-83730; Novus, Littleton, USA) and TNF-α (cat. no. DTA00C; R&D Systems), according to the manufacturer’s instructions. Cell culture supernatants were collected at 48, 60 and 72 hours after stimulation with cytokines. The volume of culture supernatant collected from each well was 2 ml. Culture supernatants were immediately centrifuged at 1500 rpm for 10 minutes at 4°C to remove the cells and were stored at -80°C until examination. The concentrations of soluble RANKL and OPG in the culture medium of chondrocytes were measured using ELISA kits for RANKL (cat. no. PK-EL-KB20452; PromoCell GmbH, Heidelberg, Germany) and OPG (cat. no. PK-EL-63750; PromoCell GmbH), according to the manufacturer’s instructions. Cell culture supernatants, without cytokine stimulation were used as the controls. Assays were performed in duplicate for each specimen, and the average values were calculated. Values less than the limit of detection were indicated as detection limit values.

### Co-culture with PBMCs and induction of osteoclastic differentiation

Normal human chondrocytes were grown to 80% confluence in CBM^TM^ with CGM^TM^ BulletKit^TM^ in a humidified incubator (ASAHI LIFE SCIENCE CO., LTD., Saitama, Japan) at 37°C and 5% CO_2_. Peripheral blood mononuclear cells (PBMCs) purchased from Precision Bioservices (Frederick, MD, USA) were cultured for 24 hours at a density of 0.8 million cells/well in 12 well-plates in α-MEM (nacalai tesque, Kyoto, Japan) supplemented with 10% fetal bovine serum and 40 ng/ml macrophage-colony stimulating factor (M-CSF) (Pepro Tech, St. Louis, MO, USA). Afterwards, confluent chondrocytes were re-suspended in α-MEM supplemented with 10% fetal bovine serum and co-cultured with PBMCs at a density of 50,000 cells/well for 21 days in the presence of 40 ng/ml M-CSF with or without 1 ng/ml LT-α. The medium and cytokines were changed every 2 days. After 21 days of co-culture, osteoclast differentiation was evaluated by staining for tartrate-resistant acid phosphatase (TRAP) using a commercial kit (Cosmo Bio, Tokyo, Japan). Counterstaining was carried out with methyl green. Osteoclast-like cells were identified by the presence of ≥ 3 nuclei and stained red or purple. The number of osteoclast-like cells was directly counted for each field of view, and counting was normalized by total cell number. The data was expressed as the average value of four fields of view (original magnification: x200).

### Statistical analysis

The results were expressed as the mean ± standard deviation. The Mann-Whitney *U* test was used to compare the data from RA and OA samples including ELISA for synovial fluid samples, Mankin’s score and RANKL expression ratio of articular cartilage. Statistical comparisons were performed using the Student’s *t*-test for *in vitro* experiments including real-time PCR, western blot analysis, ELISA for cell culture supernatants and co-culture with PBMCs. Correlations between parameters in ELISA data of RA synovial fluid samples and in immunohistochemical evaluation of articular cartilage were examined using Spearman’s correlation coefficients. The Student’s *t*-test was used in the case that data was normally distributed, while the Mann-Whitney *U* test or Spearman’s correlation coefficients was used in the case that data was not normally distributed. All differences were considered statistically significant at a p < 0.05.

## Results

### Concentrations of LT-α and TNF-α in synovial fluid

**Fig 1:**
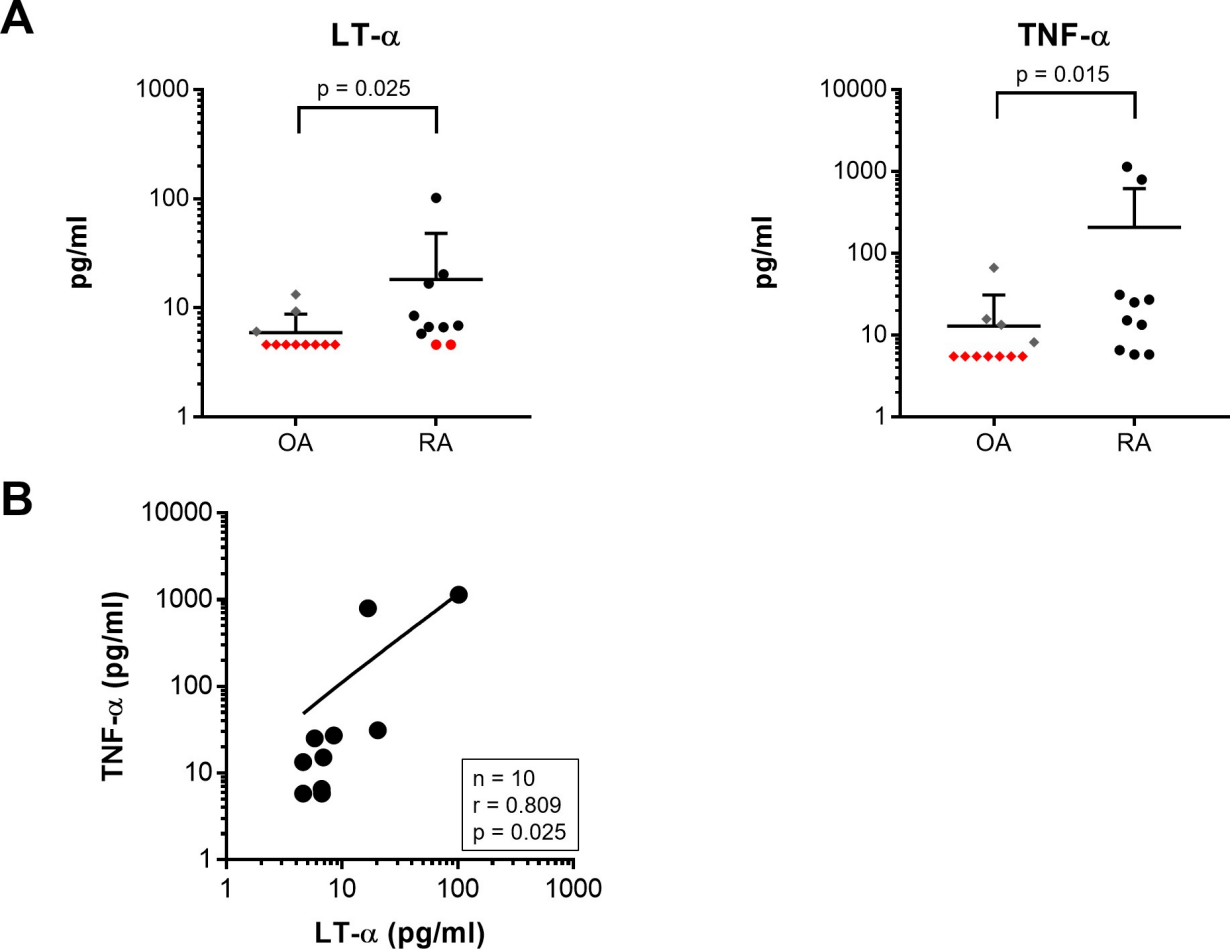
Concentrations of LT–α and TNF–α in synovial fluid. (A) Cytokine concentrations in synovial fluid collected from RA patients were higher than those in OA patients. Red points are values below the detection limit, and were taken as detection limit values. (B) A positive correlation was identified between LT–α and TNF–α concentrations in RA synovial fluid (n = 10, r = 0.809, p = 0.025).

The concentrations of LT-α and TNF-α were measured by ELISA in 10 RA and 11 OA synovial fluid samples. The detection limit of the LT-α ELISA kit was 4.6 pg/ml, and that of the TNF-α ELISA kit was 5.5 pg/ml. The values below the detection limit were taken as detection limit values. The concentration of LT-α in the synovial fluid from RA patients was 18.30 ± 28.40 (mean ± standard deviation) pg/ml, while that from OA patients was 5.96 ± 2.70 pg/ml (n = 10 in the RA group, n = 11 in the OA group). The concentration of TNF-α in synovial fluid from RA patients was 207.81 ± 390.88 pg/ml, while that from OA patients was 12.95 ± 217.32 pg/ml. The concentrations of LT-α and TNF-α were higher in RA synovial fluid than in OA synovial fluid. The concentration of LT-α was less than the detection limit in nine OA samples and two RA samples, and the concentrations of TNF-α was less than the detection limit in seven OA samples (Fig 1A). There was also a correlation between LT-α and TNF-α concentration in RA synovial fluid samples (n = 10, r = 0.809, p = 0.025) (Fig 1B).

### Immunohistochemical evaluation of RANKL in articular cartilage using optical microscopy

**Fig 2:**
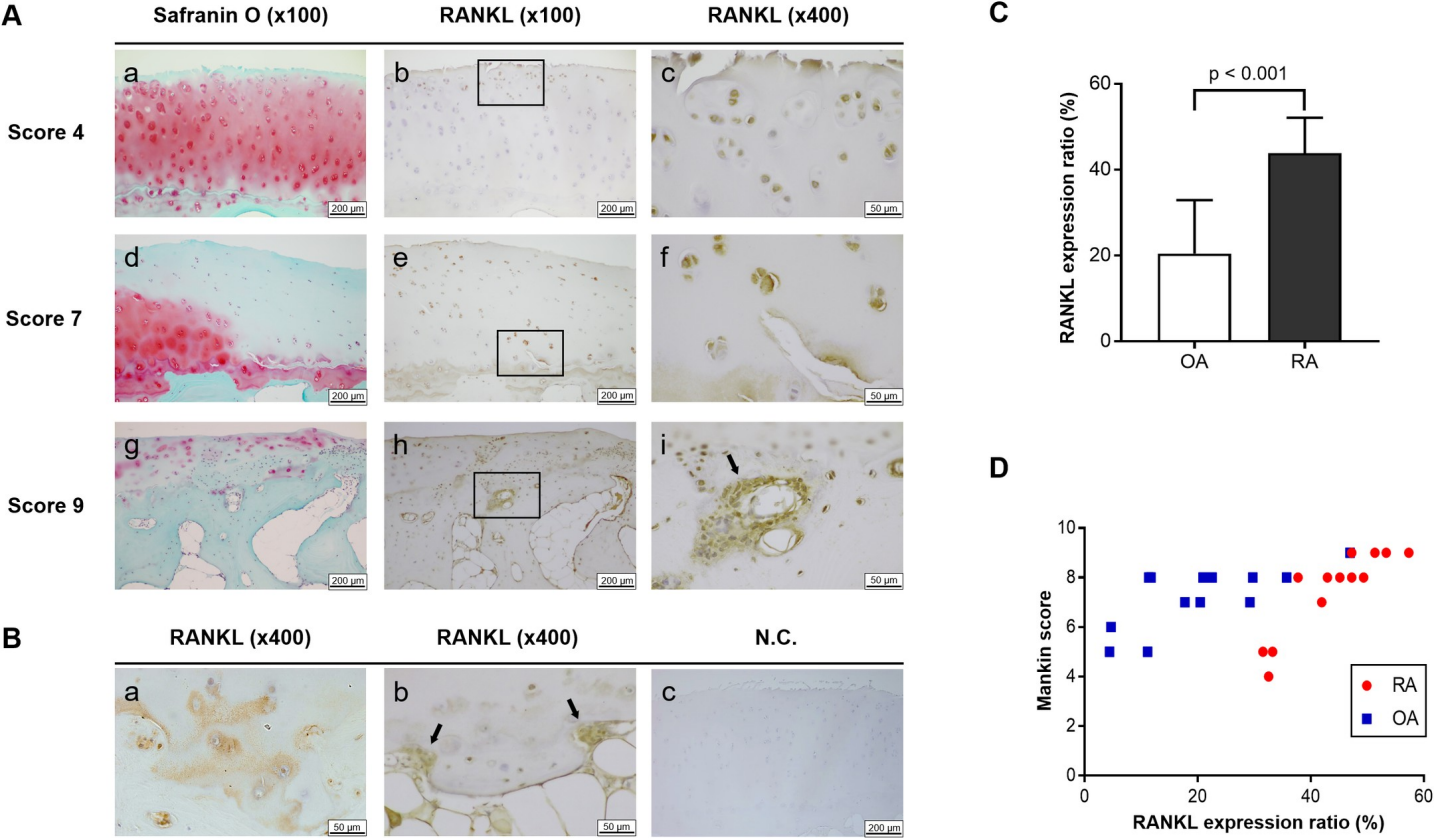
Immunohistochemical evaluation of RANKL in articular cartilage. (A) In score 4 cartilage, chondrocytes located at the superficial layer expressed RANKL (a–c). In score 7 cartilage, RANKL expression was also identified in the middle or deep layer, and extracellular RANKL was observed in the calcified layer (d–f). In score 9 cartilage, RANKL expression in chondrocytes was identified in the deep layer (g–i), especially near the blood vessels (arrow) (i). (B) Extracellular RANKL was identified in the calcified layer, especially around the chondrocytes, and diffused toward the subchondral bone (a). Multinucleated cells (arrows) were occasionally observed near the RANKL–positive chondrocytes (b). No signal was observed in the negative control (c). (C) The RANKL expression ratio of RA cartilage samples was significantly higher than that of OA cartilage samples. (D) RANKL expression ratio of RA cartilage samples positively correlated with cartilage degeneration scores (n = 13 per experimental group). N.C, negative control.

Immunohistochemical staining was performed using the 13 RA and 13 OA cartilage specimens to evaluate the expression of RANKL in articular cartilage. The extent of cartilage destruction in each specimen was evaluated using Mankin’s score. The cartilage degenerative scores did not reveal significant differences between RA samples and OA samples (p > 0.05) (Fig 2D).

In low-score RA cartilage, RANKL was mainly expressed in chondrocytes located at the superficial layer. RANKL expression in chondrocytes was also identified in the middle or deep layers, as cartilage degeneration progressed. The expression of RANKL in chondrocytes was identified in the region where cartilage degeneration was observed by Safranin O staining. The chondrocytes especially located near the newly formed blood vessels expressed RANKL (Fig 2A). Extracellular RANKL was identified in the calcified layer-especially around the chondrocytes-and diffused toward the subchondral bone. Though not that often, multinucleated cells expressing RANKL were occasionally observed near the RANKL-positive chondrocytes. No signal was observed in the negative control (Fig 2B).

The RANKL expression ratio in RA articular cartilage was approximately two-times higher than that in OA cartilage (Fig 2C), and was positively correlated with cartilage degeneration scores (n = 13, r = 0.868, p = 0.003) (Fig 2D). The RANKL expression ratio in RA cartilage specimens with tidemark integrity by the blood vessels (n = 5, 50.84 ± 4.34%) was higher than that in cartilage specimens lacking tidemark integrity (n = 8, 39.53 ± 6.41%; p = 0.016).

### Immunohistochemical evaluation of RANKL in articular cartilage using electron microscopy

**Fig 3:**
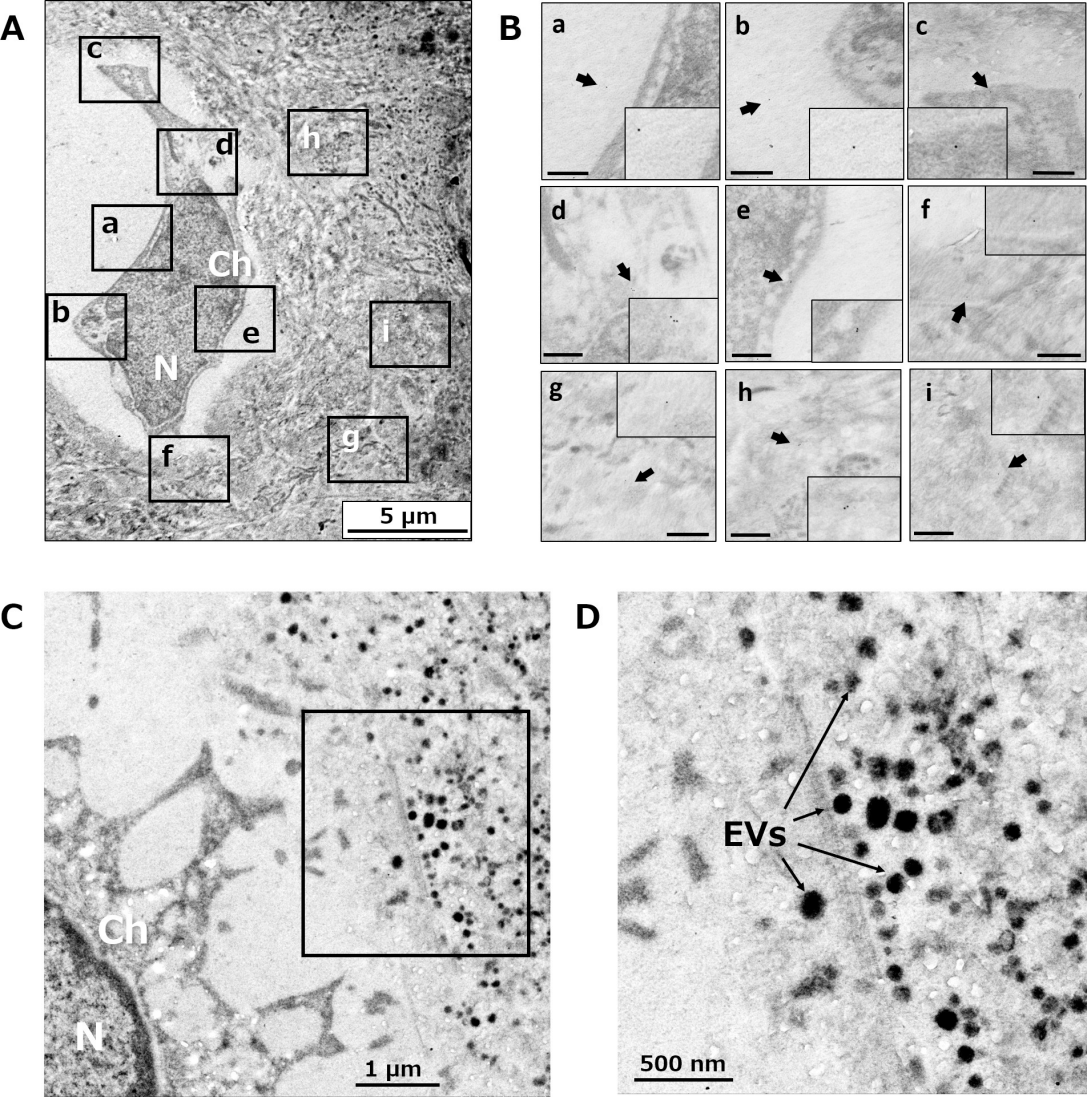
Evaluation of RANKL expression in articular cartilage by immunoelectron microscopy. The localization of 10 nm colloidal gold particles labeled RANKL antibody are observed as electron–dense punctate. (A and C) Lower magnification micrograph of deep layer chondrocyte and surrounding matrix. (B) Insets of A showing localization of immunogold at pericellular matrix (a, b), cytoplasm and cell membrane (c, d, e), and territorial matrix surrounding chondron (f, g, h, i). Scale bars = 500 nm. (D) Inset of C showing extracellular vesicles scattered randomly with the territorial matrix without labeling by colloidal gold, indicating these vesicles do not contain RANKL. Ch, chondrocyte; N, nucleus; EVs, extracellular vesicles.

Immunoelectron microscopy was also performed to examine the localization of RANKL in the deep layer and calcified layer of RA articular cartilage. The deposition of colloidal gold-labeled RANKL was detected in the extracellular space of the deep layer. RANKL was also identified inside and around the chondrocytes (Fig 3A and 3B). Those findings were consistent with the results of the immunohistochemical localization of RANKL identified using optical microscopy. However, RANKL was not observed inside the extracellular vesicles (EVs) (Fig 3C and 3D).

### Cytokines induced gene expression in chondrocytes

**Fig 4:**
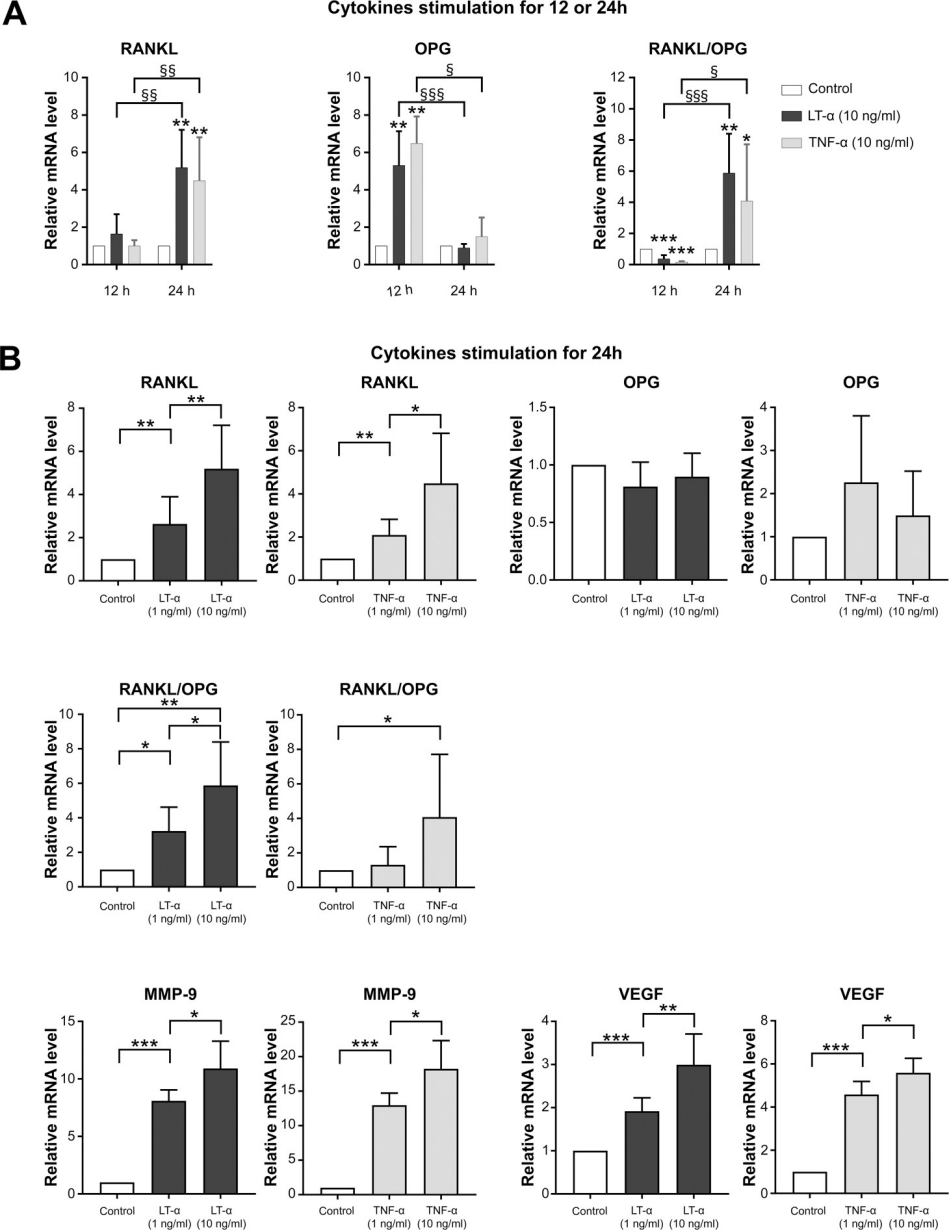
Cytokines induced gene expression in chondrocytes. (A) RANKL expression and the RANKL/OPG ratio were up–regulated after 24 hours of stimulation with LT–α or TNF–α, but not after 12 hours of stimulation. (B) RANKL expression and angiogenic factors were up–regulated in a concentration–dependent manner after 24 hours of cytokine stimulation (n = 6 per experimental group). * / § p < 0.05, ** / §§ p < 0.01, *** / §§§ p < 0.001.

To investigate the LT-α- and TNF-α-induced mRNA expression of RANKL, OPG, and angiogenic factors in chondrocytes, we performed real-time PCR. RANKL mRNA expression was up-regulated 24 hours after stimulation with both LT-α and TNF-α, but not at 12 hours. OPG mRNA expression was up-regulated after 12 hours of stimulation with both LT-α and TNF-α, but down-regulated at 24 hours after stimulation. The RANKL/OPG ratio was significantly increased at 24 hours after stimulation (Fig 4A). Therefore, we stimulated chondrocytes for 24 hours in subsequent real-time PCR experiments. Not only were RANKL levels increased in a concentration-dependent manner 24 hours after stimulation with both cytokines, but so were MMP-9, and VEGF mRNA levels (Fig 4B). OPG mRNA was not affected by LT-α and TNF-α at 24 hours; thus, resulting in a significant increase in the RNKL/OPG ratio at 24 hours after stimulation (Fig 4B).

**Fig 5:**
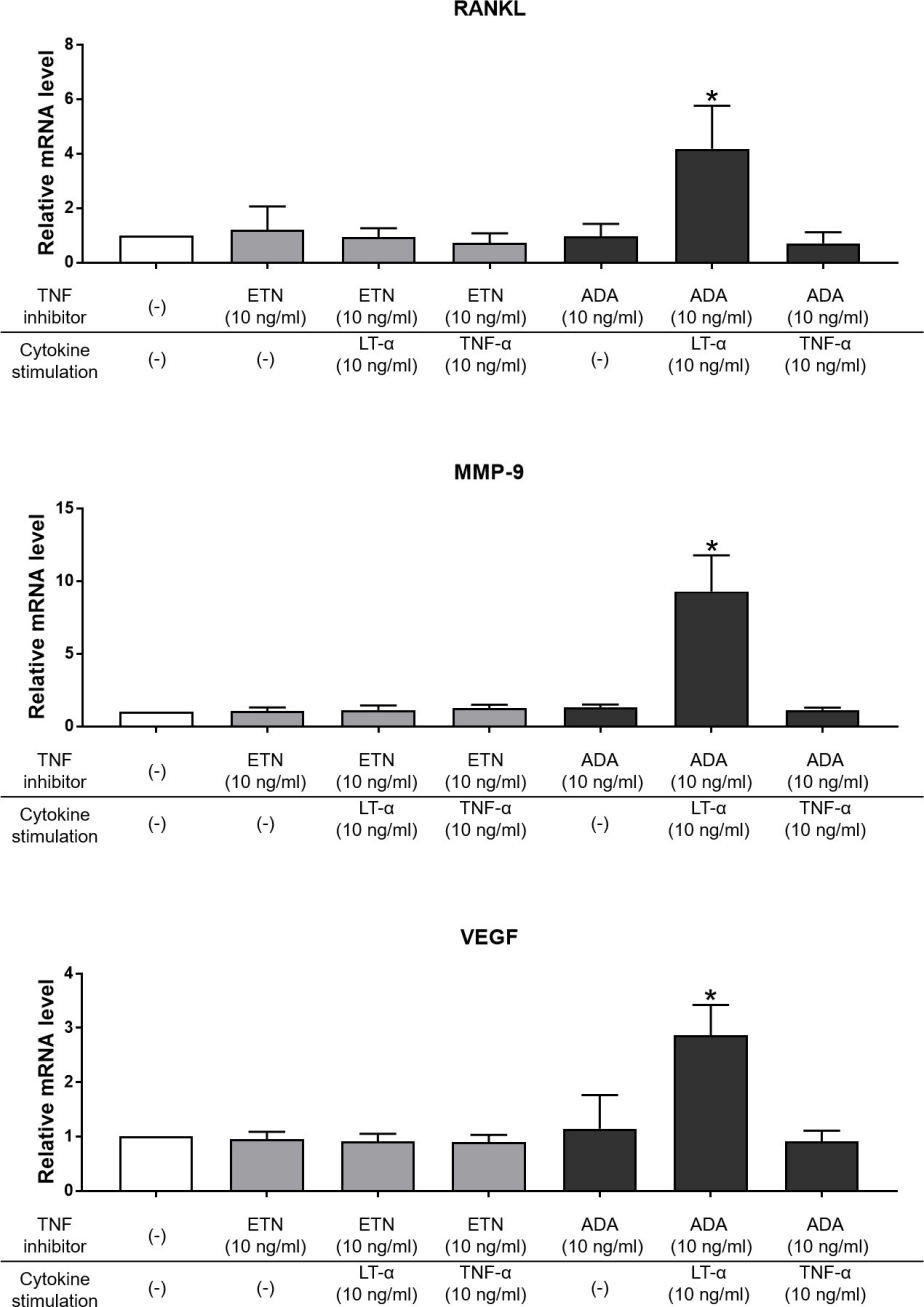
Relative mRNA level of RANKL, MMP–9 and VEGF, preincubated with TNF inhibitor before 24 hours cytokine stimulation. Each value obtained for the control cells (chondrocytes without cytokine stimulation and TNF inhibitor treatment) was set to one. Etanercept inhibited the effects of both LT–α and TNF–α, while adalimumab only inhibited the effects of TNF–α in chondrocytes. LT–α promoted the expression of RANKL, MMP–9 and VEGF independently of TNF–α (n = 6 per experimental group). * p < 0.001. ETN, etanercept; ADA, adalimumab.

Chondrocytes were preincubated for 30 minutes with 10 ng/ml etanercept or adalimumab prior to cytokines stimulation to confirm the effects of LT-α or TNF-α on the mRNA levels of RANKL, VEGF, and MMP-9. Etanercept inhibited the effects of both cytokines, while adalimumab only inhibited the effects of TNF-α in chondrocytes (Fig 5).

### Cytokines induced RANKL and OPG protein expression in chondrocytes

**Fig 6:**
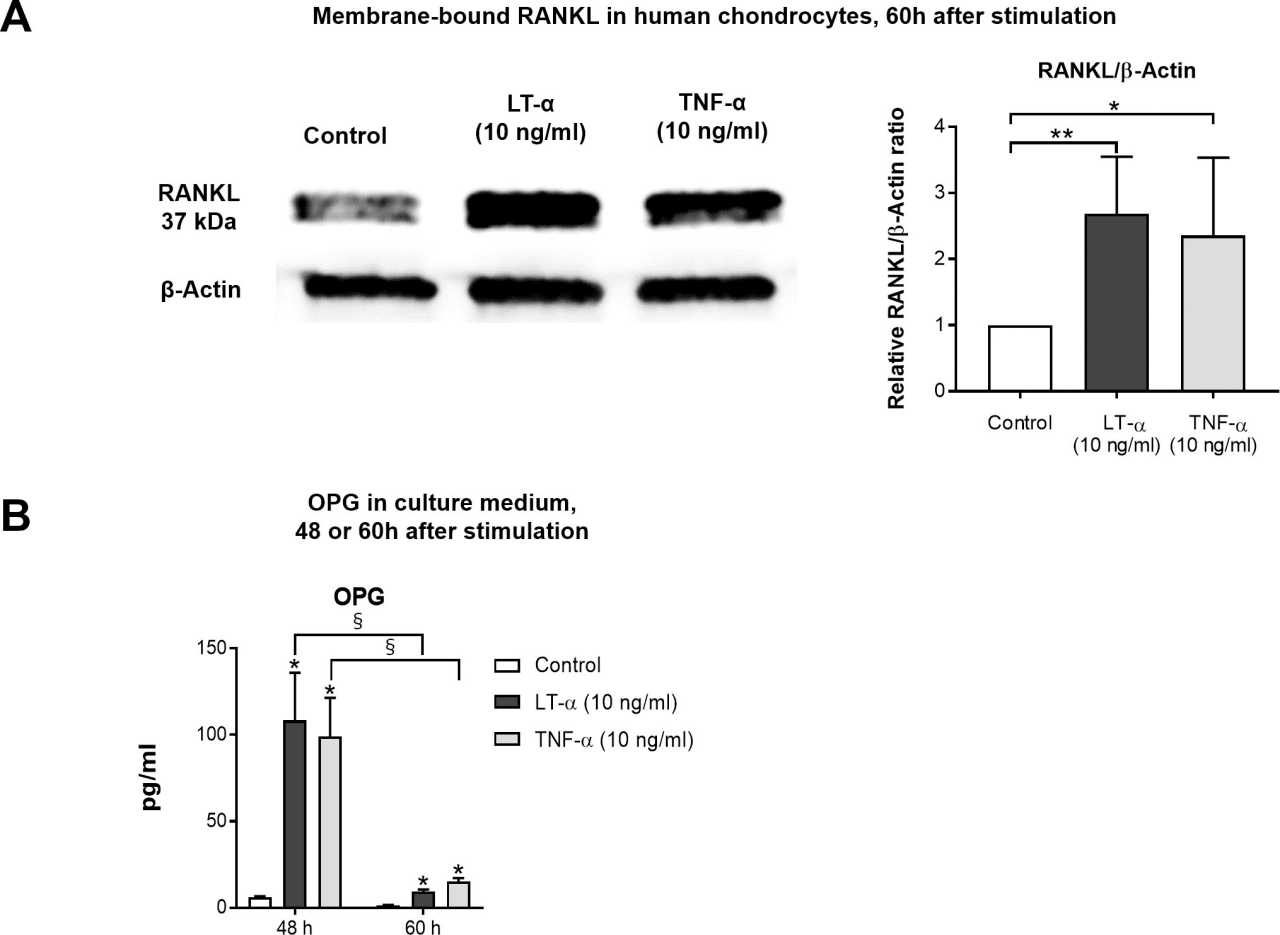
Cytokines induced the expression of RANKL and OPG in human chondrocytes. (A) Membrane–bound RANKL in cells was up–regulated 2.7–fold or 2.4–fold at 60 hours after stimulation with LT–α or TNF–α respectively (n = 5 per experiment group). * p < 0.05, ** p < 0.01. (B) OPG concentrations in culture medium were increased at 48 and 60 hours after stimulation with LT–α or TNF–α compared to the control. However, OPG concentrations in culture medium at 60 hours were markedly decreased compared to those at 48 hours. (n = 6 per experimental group). * /§ p < 0.001.

There are two major forms of RANKL: membrane-bound and soluble. Soluble RANKL results from the shedding of membrane-bound RANKL, which can be induced by various enzymes [21]. Membrane-bound RANKL expression was examined in chondrocytes by western blot analysis 60 hours after stimulation with 10 ng/ml LT-α or 10 ng/ml TNF-α. Both cytokines significantly increased membrane-bound RANKL expression (Fig 6A).

Soluble RANKL expression was determined in culture medium using ELISAs 60 hours after stimulation with 10 ng/ml LT-α or 10 ng/ml TNF-α. Neither cytokine increased soluble RANKL expression. We also examined the culture medium at 48 or 72 hours after stimulation, considering the possibility that the timing of expression differed between the membrane-bound and soluble forms. However, soluble RANKL expression did not increase compared to the control at any time point (S1 Table).

OPG protein expression in culture medium was determined by ELISA at 48 or 60 hours after cytokine stimulation. OPG concentrations were increased at 48 and 60 hours after stimulation, compared to control, however, OPG concentrations at 60 hours were markedly decreased compared to expression at 48 hours (Fig 6B). Thus, the collective findings suggested that membrane-bound RANKL expression increased at 60 hours after stimulation, while OPG expression was decreased.

### RANKL derived from chondrocytes promoted the differentiation of PBMCs into osteoclast-like cells

**Fig 7:**
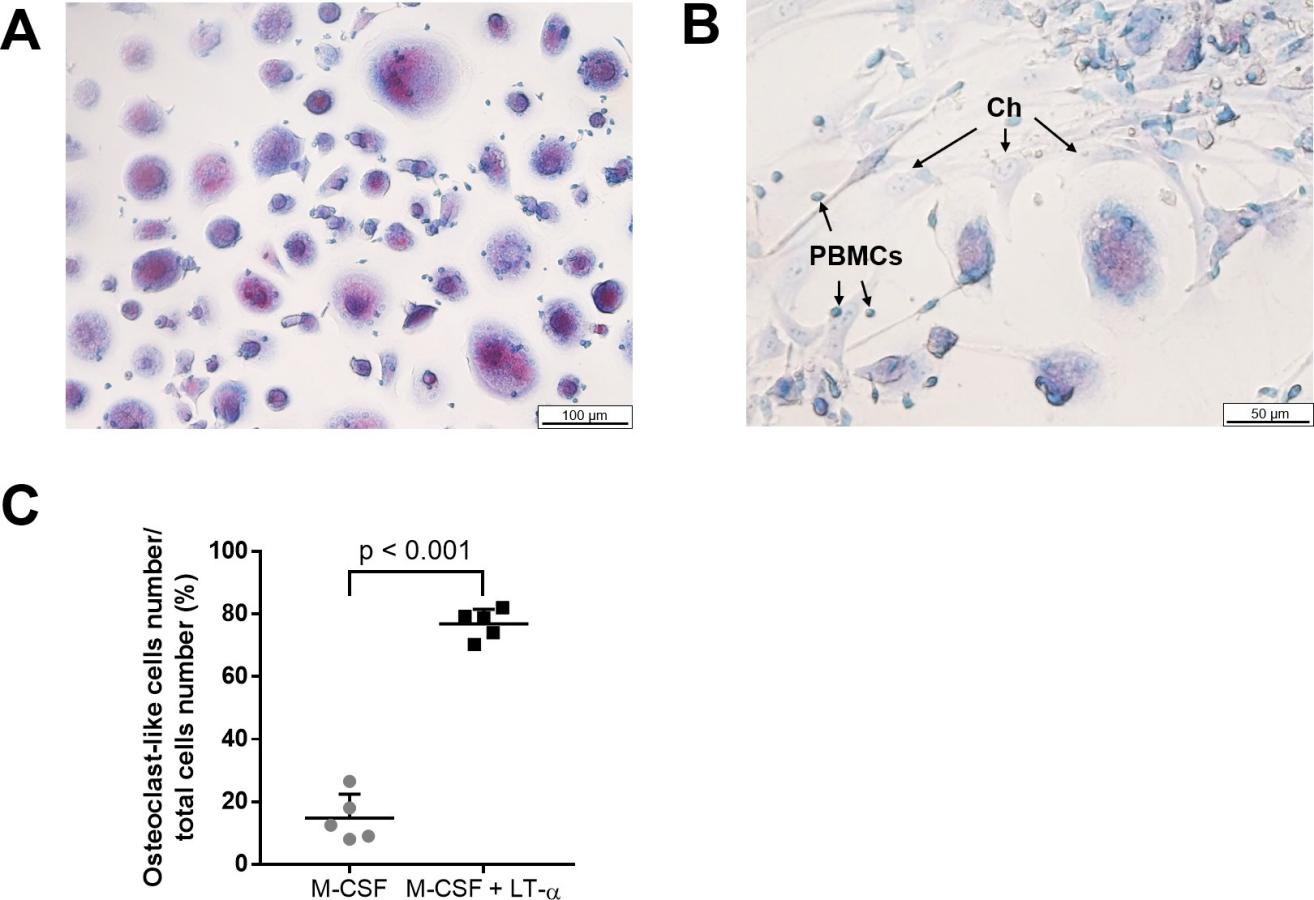
RANKL synthesized by human chondrocytes promoted the differentiation of osteoclast–like cells. (A) RANKL produced by chondrocytes stimulated with LT–α 1 ng/ml induced the differentiation of monocytes into osteoclast–like cells. (B) Although TRAP–positive multinucleated cells were observed in the absence of LT–α, their TRAP staining was weak. The direct contact between chondrocytes and osteoclast–like cells or PBMCs was observed. (C) The number of TRAP–positive multinucleated cell in presence of LT–α was 5.2–hold larger than that in the absence of LT–α (n = 5 per experimental group). Ch, chondrocyte; PBMCs, Peripheral blood mononuclear cells.

To investigate whether RANKL produced by LT-α-stimulated chondrocytes had osteoclastogenic activity, PBMCs with normal human articular chondrocytes were co-cultured in α-MEM containing 10% fetal bovine serum and 40 ng/ml M-CSF in the presence or absence of 1 ng/ml LT-α. RANKL produced by LT-α-stimulated chondrocytes induced the differentiation of monocytes into osteoclast-like cells (Fig 7A). Although TRAP-positive multinucleated cells were observed in the absence of LT-α, dyeability was weak and its color was slightly purple (Fig 7B). Additionally, the number of TRAP-positive multinucleated cell was significantly greater than that in the absence of LT-α (Fig 7C). In the presence of LT- α, few chondrocytes were found around osteoclast-like cells (Fig 7A), while in the absence of LT- α, some chondrocytes still survived, and direct contact of chondrocytes with osteoclast-like cells or PBMCs was observed (Fig 7B).

## Discussion

The histopathological findings of RA cartilage often show substantial cartilage erosion by cells and blood vessels from the subchondral bone region, as well as cartilage direct invasion by synovial pannus. It has been traditionally described as “bidirectional cartilage erosion” [22]. More recently, the trans-osseous route, direct damage of cartilage by pannus, and the release of proteolytic enzymes by synovial tissues has been proposed as the “third mechanism” of cartilage destruction in RA patients [23]. The elucidation of the mechanism of focal bone loss in RA developed rapidly after year 2000, with reports of the accumulation of bone-resorbing osteoclasts at the bone-pannus interface, and RANKL expression at the mRNA and protein levels in synovial tissue from RA patients [24]. The cells responsible for RANKL expression in the synovial tissue were synovial fibroblasts [25–29] and activated T cells [30–33]. Some reports observed the emergence of osteoclasts or preosteoclasts in the bone marrow or at the subchondral bone erosion [25, 26, 29], and those cells were later revealed to be involved in the pathogenesis of MRI-detected bone marrow edema in RA patients [34]. However, the mechanisms underlying how those cells were recruited to the cartilage-bone interface, and directed to differentiate into osteoclasts have not been fully determined.

RANKL expression in chondrocytes was first described in the pre-hypertrophic and hypertrophic chondrocytes in embryonic mouse long bones [10]. In the arthritic joints, the expressions of RANK, RANKL and OPG expression was demonstrated in the human OA cartilage [11]. Although RANKL expression extended throughout the midzone of the cartilage and included clusters of proliferating OA chondrocytes, RANKL did not induce proinflammatory mediators and was not directly involved in cartilage degradation during arthritis. Thus, those results suggest that RANKL derived from OA chondrocyte might have a role in the recruitment or activation of other cell types within the joint. Tat *et al*. examined the expression of OPG, RANK, and RANKL in human normal and OA chondrocytes, and observed reduced OPG/RANKL ratios in OA chondrocytes. That study also found that IL-1β, TNF-α, and PGE_2_ significantly enhanced the expression of OPG and RANKL [14].

In AIA rabbits, RANKL expression was observed throughout all cartilage zones and was especially increased in the calcified cartilage [13]. In the first part of the current study, we investigated the RANKL expression in articular cartilage from human RA patients, and found that it was higher than that in OA patients, and positively correlated to the severity of cartilage damage, as determined by Mankin’s score. Those results further confirmed that chondrocyte-derived RANKL might be involved in cartilage degeneration in RA.

RA synovial tissue produce a variety of cytokines that promote RANKL expression, including TNF-α, IL-1, IL-6, M-CSF, and IL-17. However, no information exists on the effect of LT-α on RANKL expression in chondrocytes. It has been reported that the levels of LT-α are increased in the serum and synovial tissue from patients with RA compared to healthy controls or patients with OA [35, 36]. In the latter part of the current study, we demonstrated for the first time that LT-α is as effective as TNF-α in stimulating chondrocytes to express RANKL. Although both TNF-α and LT-α promoted the expression of OPG 12 hours after stimulation, the RANKL/OPG ratio increased in a dose-dependent manner 24 hours after stimulation. In addition to the increase in RANKL/OPG ratio, LT-α activated NF-κB and induced the expression of IL-6, IL-8, and MMP-3 in FLS from RA patients [37]. Although these findings indicated that blocking LT-α may be of potential benefit in specific population of patients with RA who do not respond to anti-TNF-α therapy or develop resistance to such therapy, they should be further verified with experimental model or clinical data.

The role of RANKL expressed by chondrocyte remains unclear in the pathogenesis of RA. Using AIA rabbits, Martinez-Calatrava *et al*. demonstrated that about 30% of the total RANKL in the arthritic joints was synthesized by articular cartilage, and that increased RANKL/OPG ratios were associated with subchondral bone loss [13]. Petit *et al*. reported that cartilage damage was less severe in the serum transfer model of arthritis created in RANKL knockout mice [38]

It has been reported that RANKL synthesized by PGE_2_-stimulated chondrocytes is biologically active and is responsible for the osteoclast differentiation of PBMCs into osteoclasts [13]. In the current study, whether chondrocytes expressed RANKL in response to LT-α stimulation promoted osteoclast differentiation was verified by co-culturing chondrocytes with PBMCs. We showed that RANKL produced by human chondrocytes stimulated by LT-α exhibited biological activity to promote PBMCs to differentiate into osteoclast-like cells in the absence of exogenous RANKL.

Activation of osteoclastogenesis in the bone marrow might induce the vulnerability and subsequent loss of function as a shock-absorber of the subchondral bone plate and calcified cartilage [22]. However, chondrocytes surrounded by the cartilage matrix did not appear to make direct contact with bone marrow cells without cartilage invasion of the blood vessels. In this study, we demostrated that LT-α and TNF-α promoted VEGF and MMP-9 gene expression in human chondrocytes. Therefore, both cytokines might be involved in vascular invasion of articular cartilage from the bone marrow, as well as promote precursor and mature osteoclast interaction with chondrocytes [39].

RANKL has been shown to increase vascular permeability and could regulate angiogenesis and endothelial cell function [40, 41]. In addition, RANKL was also a chemotactic factor for monocytes [42]. In the current study, RANKL expression in chondrocytes from RA patients was higher in the areas with vascular invasion and tidemark integrity than in the areas without vascular invasion. In addition, cartilage erosion by multinucleated cells from the subchondral region was occasionally observed near the chondrocytes expressing RANKL. Thus, the collective findings suggest that RANKL can promote the contiguity of osteoclast precursors recruited through blood vessels to chondrocytes.

In this study, the amount of OPG in the culture supernatant decreased at 60 hours after cytokine stimulation, and no increase in soluble RANKL in the culture supernatant was observed. It was reported that upregulation of RANKL was associated with downregulation of OPG, or at least lower induction of OPG [43]. In our study, the expression of RANKL mRNA was increased at 24 hours after stimulation, while OPG mRNA expression was decreased. The expression of membrane-bound RANKL in cells increased at 60 hours after stimulation, while OPG expression in culture supernatant was decreased. We speculated that OPG may have decreased at 60 hours after stimulation due to bounding with soluble RANKL in the culture medium which was derived by the shedding of membrane-bound RANKL.

In accordance with prior studies, extracellular RANKL was most frequently located surrounding the hypertrophic chondrocytes in the calcified layer [13, 44]. We have also shown that extracellular RANKL was identified around the chondrocytes in the calcified layer, exhibiting diffuse distribution toward the subchondral bone. However, how soluble RANKL released by chondrocytes affects bone marrow cells remains unknown.

Extracellular Vesicles (EVs) play important roles in cell-to-cell communication by transporting various proteins, microRNAs, and mRNAs [45]. It has recently been suggested that EVs from mature osteoclasts contained RANK, and RANK in EVs may competitively inhibit the function of RANK on osteoclast surfaces by binding to RANKL [46]. Therefore, we hypothesized that membrane-bound RANKL encapsulated in EVs diffuse to subchondral bone. However, immunoelectron microscopy revealed that RANKL signals were observed in chondrocytes and the extracellular matrix, whereas it could not be confirmed in EVs around the calcified cartilage. Pan *et al*. suggested that solute transport between subchondral bone and articular cartilage occurred through permeable calcified cartilage, and small nutrients and signaling molecules with molecular weights less than 376 kDa may be able to perfuse over tissues [47]. As the molecular weight of non-glycosylated soluble RANKL is ~18 kDa, chondrocyte-derived soluble RANKL is permeable through calcified cartilage. Nevertheless, whether soluble RANKL promotes osteoclastogenesis remains unknown.

A recent report demonstrated that membrane-bound RANKL was sufficient for the most functions of this protein such as osteoclast formation, and immune cell and mammary gland development, whereas soluble RANKL does contributed to physiological bone remodeling in adult mice [48]. Although it is possible that soluble RANKL might play a role in the communication of chondrocytes with osteoclast progenitor cells at the cartilage-bone interface, the elucidation of the precise role of soluble RANKL requires further investigation.

This study focused on chondrocytes, while osteoblasts are involved in osteoclast regulation by expressing RANKL [49], and communication between osteoblasts and osteoclasts via direct contact, cytokines, and extracellular matrix interaction, regulates the bone cell biology [50]. In a study of murine RA model using dynamic bone histomorphometry, bone formation rates at bone surfaces adjacent to inflammation were similar to those observed in non-arthritic bone, suggesting that osteoblasts are not able to compensate for the increased bone resorption [51]. RANKL derived from chondrocytes might affect crosstalk between osteoblasts and osteoclasts, and might cause an imbalance between bone formation and bone resorption.

There were several limitations to this study. First, the main limitation was the number of clinical samples, and it was not possible to obtain more samples during the recruitment period. Second, though we collected RA samples including synovial fluid and articular cartilage from patients who had not received TNF inhibitors, RA samples used in this study might be affected by drugs other than TNF inhibitors. Third, the inflammatory condition within the RA joint is complicated, and factors other than LT- α or TNF- α stimulation were not examined in the current *in vitro* model.

In conclusion, we demonstrated that chondrocytes in RA cartilage express relatively higher amounts of RANKL than chondrocytes in OA cartilage, and its expression was associated with cartilage degeneration. LT-α, a close homolog of TNF-α, promoted RANKL expression in chondrocytes with an induction equivalent to that obtained using TNF-α. The results of PBMCs co-culture with chondrocytes demonstrated that LT-α-induced membrane-bound RANKL in chondrocytes promoted the osteoclastic differentiation in PBMCs. Our results indicate that RANKL produced by chondrocytes may contribute to cartilage destruction during RA, and LT-α could promote the RANKL expression in human chondrocytes independently of TNF-α.

## Supporting information

S1 Table LT-α and TNF-α failed to promote soluble RANKL release to the cultured medium of chondrocytes. (DOCX)

S1 Raw images (PDF)
