# Supplementary material for: RANKL expression in chondrocytes and its promotion by lymphotoxin-α in the course of cartilage destruction during rheumatoid arthritis
Source: PLoS One. 2021 Jul 7;16(7):e0254268. doi: 10.1371/journal.pone.0254268 (PMC8263262; doi:10.1371/journal.pone.0254268)
Supplement: S1 Table — (DOCX) [file pone.0254268.s001.docx]

**Soluble RANKL expression in culture medium after 48 or 60, or 70 hours stimulation with LT-α or TNF-α.**

|  | **Control** | **LT-α**  **(10 ng/ml)** | **TNF-α**  **(10 ng/ml)** |
| --- | --- | --- | --- |
| **48h** | 0.41 ± 0.14 pg/ml | 0.66 ± 0.40 pg/ml | 0.40 ± 0.15 pg/ml |
| **60h** | 0.33 ± 0.13 pg/ml | 0.36 ± 0.15 pg/ml | 0.53 ± 0.09 pg/ml |
| **72h** | 0.53 ± 0.11 pg/ml | 0.46 ± 0.12 pg/ml | 0.51 ± 0.11 pg/ml |

The data represent the mean ± standard deviation (n = 6 per experimental group).
