# Supplementary material for: RANKL expression in chondrocytes and its promotion by lymphotoxin-α in the course of cartilage destruction during rheumatoid arthritis
Source: PLoS One. 2021 Jul 7;16(7):e0254268. doi: 10.1371/journal.pone.0254268 (PMC8263262; doi:10.1371/journal.pone.0254268)
Supplement: S1 Raw images — (PDF) [file pone.0254268.s002.pdf]

Membrane-bound RANKL in human chondrocytes, 60h after stimulation

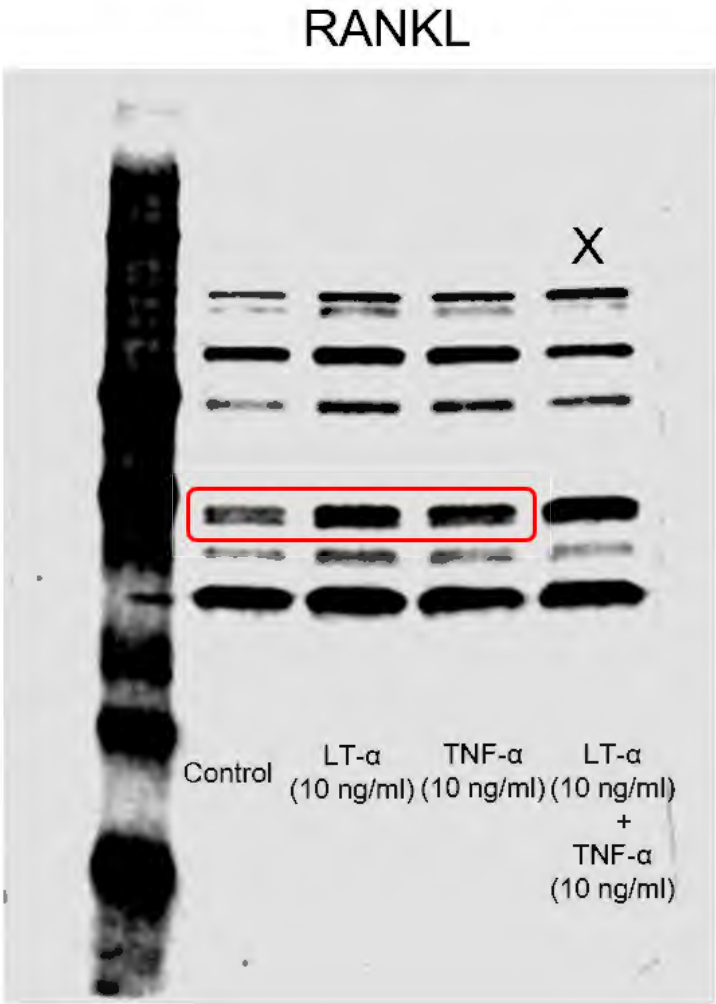

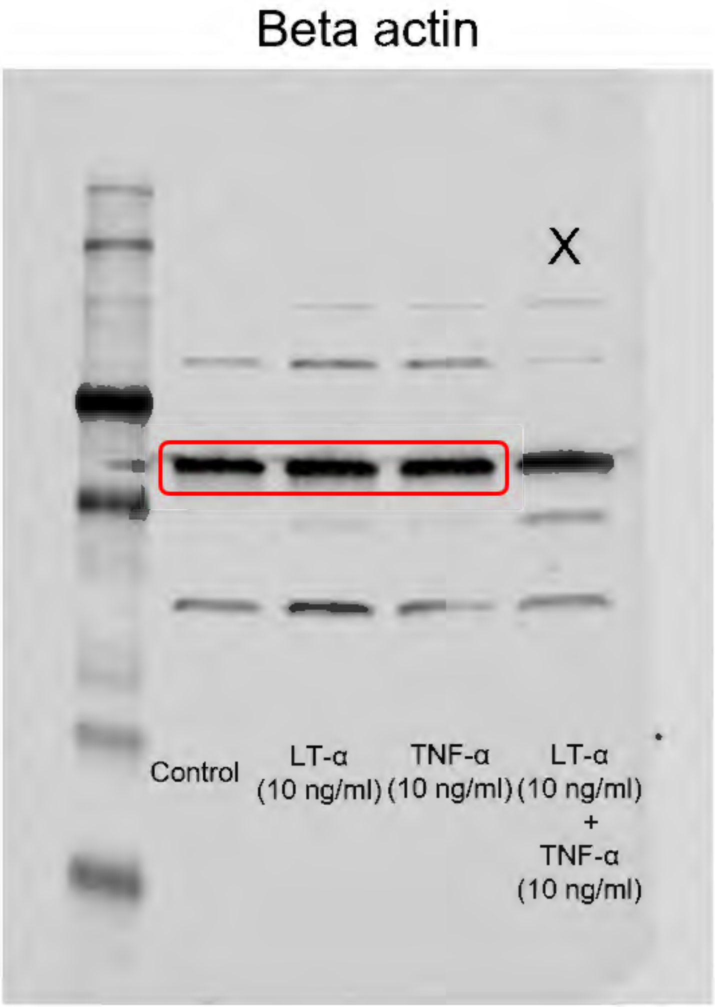

The area of red frame was captured using Microsoft snipping tool.
